# Single-cell transcriptomic analysis reveals the critical molecular pattern of UV-induced cutaneous squamous cell carcinoma

**DOI:** 10.1038/s41419-021-04477-y

**Published:** 2021-12-21

**Authors:** Guorong Yan, Liang Li, Sibo Zhu, Yuhao Wu, Yeqiang Liu, Lude Zhu, Zijun Zhao, Fei Wu, Ning Jia, Caihe Liao, Long Jiang, Qingyu Zeng, Peiru Wang, Lei Shi, Zhe Zheng, Shan Fang, Guolong Zhang, Yichen Tang, Xiuli Wang

**Affiliations:** 1 Institute of Photomedicine, Shanghai Skin Disease Hospital, School of Medicine, Tongji University, Shanghai, 200092 China; 2 Department of Dermatologic Surgery, Shanghai Skin Disease Hospital, School of Medicine, Tongji University, Shanghai, 200092 China; 3 State Key Laboratory of Genetic Engineering, School of Life Sciences, Fudan University, Shanghai, 200438 China; 4 Department of Pathology, Shanghai Skin Disease Hospital, School of Medicine, Tongji University, Shanghai, 200092 China

**Keywords:** Squamous cell carcinoma, Oncogenesis

## Abstract

Cutaneous squamous cell carcinoma (cSCC) is the second most common nonmelanoma skin cancer characterized by high invasiveness, heterogeneity, and mainly occurs in the ultraviolet (UV)-exposed regions of the skin, but its pathogenesis is still unclear. Here, we generated single-cell transcriptome profiles for 350 cells from six primary UV-induced cSCCs, together with matched adjacent skin samples, and three healthy control skin tissues by single-cell RNA-sequencing technology based on Smart-seq2 strategy. A series of bioinformatics analyses and in vitro experiments were used to decipher and validate the critical molecular pattern of cSCC. Results showed that cSCC cells and normal keratinocytes were significantly distinct in gene expression and chromosomal copy number variation. Furthermore, cSCC cells exhibited 18 hallmark pathways of cancer by gene set enrichment analysis. Differential expression analysis demonstrated that many members belonging to S100 gene family, SPRR gene family, and *FABP5* were significantly upregulated in cSCC cells. Further experiments confirmed their upregulation and showed that *S100A9* or *FABP5* knockdown in cSCC cells inhibited their proliferation and migration through NF-κB pathway. Taken together, our data provide a valuable resource for deciphering the molecular pattern in UV-induced cSCC at a single-cell level and suggest that *S100A9* and *FABP5* may provide novel targets for therapeutic intervention of cSCC in the future.

## Introduction

The impact of cutaneous squamous cell carcinoma (cSCC) in a global context is monstrous and cannot be ignored. According to the lasted global burden of disease study, nonmelanoma skin cancer (NMSC) was the most prevalent cancer in both men and women in 2017, reaching 7.7 million incident cases, of which 5.9 million were caused by basal cell carcinoma and 1.8 million due to cSCC [1]. Furthermore, another recent global cancer statistic research also estimated that there were almost 1.2 million incident cases of cSCC in 2020 [2]. As the second most common NMSC, cSCC develops preferentially in the interfollicular epidermis since the unrestricted proliferation of epidermal keratinocytes (KCs). Cumulative exposure to ultraviolet (UV) radiation and carcinogenic chemicals, the use of immunosuppressive agents, and genetic susceptibility were extensively considered as the major risk factors of cSCC [3]. Although cSCC exhibits a relatively low risk of lymphatic metastasis and good prognosis, there were still 2.1–5% of cSCCs who progressed into metastases [4, 5], ultimately causing 3900–8800 deaths in the US [3], accounting for 20% of cutaneous carcinoma-related deaths [6]. Recently, two approved anti-programmed death protein-1 (PD-1) antibodies, including Cemiplimab and Pembrolizumab used for the treatment of locally advanced and metastatic cSCC, are promising. Still, only 34–50% objective response rate was obtained [7–9], emphasizing the need for the better characterization of cSCC to explore novel therapeutic targets.

Gene expression profiles of tumor tissues have revolutionized our understandings on cSCC [10]. Yet most analyses of gene expression are dominated by bulk RNA-sequencing (RNA-seq) technology, in which the expression profile of each gene is profiled by the average expression of all sequenced cells for each biological sample, masking many critical aspects of intra-tumor heterogeneity (ITH) and hindering our understandings of cSCC biology. What is exciting is that the emergence of single-cell RNA-sequencing (scRNA-seq) technology has revealed major cellular components, heterogeneity of human normal skin, and a wide range of human skin diseases at the single-cell transcriptome level [11], and has been used for the identification of cell type-specific expression quantitative trait loci [12]. Miao et al. found that tumor-initiating stem cells can selectively acquire CD80 by the cSCC mouse model using scRNA-seq, which directly dampen the cytotoxic T cell activity, implicating the vital role of tumor-initiating stem cells in activating the immune checkpoint therapy [13]. Yost et al. performed scRNA-seq in the cSCC patients before and after anti-PD-1 therapy and observed that T cell response to checkpoint blockade relies on the recruitment of novel T cells [14]. Moreover, a recent study identified a tumor-specific KC population in cSCC using scRNA-seq, which acted as a hub for intercellular communications [15]. However, most studies employed a 10X Genomics Chromium-based scRNA-seq strategy, which exhibits the advantage in the huge sequenced cell number, but biases the expression quantifications in some key driver genes, when compared it with the Smart-seq2 scRNA-seq strategy. Besides, although the studied tissues were paired for cSCC tissue and adjacent skin from Ji et al., not all cSCC tissues were collected from UV-exposed sites, which may also complicate the interpretation of the result. Furthermore, different ethnic backgrounds tend to possess some specific genetic architectures, and they did not focus on the specific gene expression profile in the cSCC cells. Therefore, it is still vital to decipher the key driver genes of cSCC by a high accuracy scRNA-seq technology in different races.

Here, we aimed to adopt the Smart-seq2-based scRNA-seq approach to dissect the cSCC ITH and critical dysregulated genes in promoting cSCC progression. A total of 350 quality-controlled single-cell transcriptomes and 5 cell populations were obtained from six primary human cSCCs and patient-matched skin. A wide range of known and novel dysregulated genes, including the S100 gene family, SPRR gene family, and *FABP5* associated with cSCC progression, were sought out by bioinformatics analyses and validated by a series of in vitro cell experiments. Thus, our findings will update the understanding of the mechanism of UV-induced cSCC progression.

## Materials and methods

### Human patient samples

Primary cSCC and patient-matched normal adjacent skin samples were obtained from six consecutive cSCC patients (86 ± 6 years), all of whom were immunotherapy-naive. The detailed clinico-pathological characteristics of these patients are presented in Table 1. All diagnoses of cSCC were verified histologically by a board-certified dermatopathologist (Supplementary Fig. S1), and human papilloma virus-negative. In addition, three more normal young skin tissues were obtained from donors (28 ± 5 years) who underwent resection surgery, for the investigation of KCs between the young and the old. In specific, all fresh specimens for experiments were collected during surgical resection and immediately stored in RPMI with 10% fetal bovine serum, 1% amphotericin B, and 1% penicillin/streptomycin. In addition, paired-samples were also fixed in 10% formalin solution for downstream immunohistochemistry (IHC) analysis.

**Table 1:** Clinico-pathological characteristics of the patient cohort.

Patient	Gender	Age (years)	Location	Differentiation	Immune-infiltration	TNM stage
1	F	85	Right cheek	Well differentiated	High	T1cN0M0
2	F	76	Forehead	Well differentiated	Moderate	T1cN0M0
3	M	89	Crown of scalp	Moderately differentiated	High	T3N0M0
4	F	88	Left cheek	Moderately differentiated	High	T1bN0M0
5	F	91	Right cheek	Well differentiated	High	T3N1M0
6	M	91	Right temple	Well differentiated	High	TisN0M0

### Tissue dissociation

Fresh skin biopsy samples including primary cSCC and patient-matched normal skin were minced. Each tissue was dissociated in a 2 ml tube (Axygen, China) containing 1 ml pre-warmed M199-media (ThermoFisher Scientific, USA), 2 mg/ml collagenase P (Roche, USA), and 10 U/μl DNase I (Roche) according to the protocol described by Tirosh et al. [16]. Tissues were digested for 60 min at 37 °C and then pipetted up and down every 10 min for 10 times. After initial isolation, cells were filtered by a 70 μm nylon mesh (ThermoFisher Scientific) and were spun at 450 × g for 5 min to yield single-cell suspensions. Pellet was resuspended for live cell staining using carboxyfluorescein diacetate succinimidyl ester incubation for 5 min.

### Single-cell cDNA library construction and sequencing

Single cells from each sample were manually and randomly picked by mouth pipette under fluorescent microscopy (X71, Olympus, Japan) and then transferred into 0.2 ml PCR tubes containing 2 μl cell lysis buffer. Libraries of isolated single cells were constructed following the Smart-seq2 protocol [17] with only modifications on the reverse transcription and amplification cycle.

Oligo-dT primed mRNA reverse transcription was performed with SMARTScribe reverse transcriptase (Takara, Japan) and locked by template-switching oligonucleotides (Exiqon, Denmark). After that, full-length cDNA amplification was conducted by 22 cycles of PCR amplification with HiFi-HotStart ReadyMix (KAPA Biosystems, USA) and subsequent 0.6× AMPure beads purification (BD, USA). Barcoded libraries were fragmented and tagmented with Nextera XT Library Prep kit (Illumina). Pooled libraries with unique N5-N7 barcodes were sequenced using a Hiseq 2500 platform (Illumina) with a single-end 50 base-pair length.

### scRNA-seq data processing

Raw fastq data of scRNA-seq were first assessed by FastQC (v0.11.9) [18]. Then, the reads with the adaptor or poly-A sequences were removed from the raw fastq data before alignment using Trimmomatic (v0.36) [19]. The low-quality reads with “N” bases rate >0.05, average quality <20, and the read length <30 bp were also removed. Second, clean reads were aligned to the UCSC human reference genome hg19 using HiSat2 (v2.0.5) [20]. We used FeatureCounts [21] to count the number of uniquely mapped read pairs for each gene. We defined gene with count >1 as the detected gene. Low-quality cells (<1000 genes/cell, <10,000 library size/cell, and >10% mitochondrial genes) were excluded. Then, we normalized the gene expression by computerSumFactors and logNormCounts functions using scran R-package [22].

### Cell type identification

The Seurat R-package was employed to identify the major cell types [23]. A total of 2000 highly variable genes were sought out using the FindVariableGenes function and then used to perform principal component analysis (PCA) for the dimensionality reduction. Significant principal components (PCs) were identified using the JackStraw function. PCs 1–14 were used for graph-based clustering (resolution = 1) to identify distinct clusters. These clusters were visualized by t-distributed stochastic neighbor embedding (t-SNE) analysis using previously computed PCs 1–14. The cell markers were determined by the FindAllMarkers function with 0.1 and 0.25 for parameters of min.pct and logfc.threshold, respectively. Then, we annotated the identity of each cell cluster based on the expression of known markers: *KRT15*, *KRT7* (normal KCs), *CFD*, *APOD* (fibroblasts (FBs)), *CDKN2A*, *KRT6A* (cSCCs), *CD79A*, *IGLL1*, *JCHAIN*, *MZB1* (B cells), and *RGS1*, *TYROBP* (dendritic cells (DCs)).

### CNV inference

To investigate the copy number variation (CNV) level in normal KCs and cSCC cells, we calculated CNVs in cluster 0 and cluster 2 based on the original gene count data of scRNA-seq data using inferCNV R-package (v.1.2.1). The normal KCs from cluster 0 were calculated as a CNV control to eliminate the individual somatic CNV. The CNV score was computed on a moving average window equal to 101, and the cutoff parameter was set to 1. The scores were restricted to the range of −1 to 1 by replacing all values >1 with 1 and all values <−1 with −1.

### DEGs identification and function annotation

To detect the differentially expressed genes (DEGs) between KCs and cSCC cells, we used the FindMarkers function implemented in Seurat R-package. The threshold for DEGs detection was as follows: the absolute value of average logFC was set to 1 and the adjusted *P* value was set to 0.05. Then, Gene Ontology (GO) analysis at the biological process level, Kyoto Encyclopedia of Genes and Genomes (KEGG) analysis, and protein–protein interaction (PPI) network analysis were performed in the STRING website (https://www.string-db.org). Gene set enrichment analysis (GSEA) was conducted using clusterProfiler R-package.

### TCGA database analysis

In order to further investigate the expressions of *S100A9*, *SPRR2A*, and *FABP5* among different SCCs, we used the RNA-seq data including cervical squamous cell carcinoma and endocervical adenocarcinoma (CESC), esophageal carcinoma (ESCA), head and neck squamous cell carcinoma (HNSC), and lung squamous cell carcinoma (LUSC) from The Cancer Genome Atlas (TCGA) database. Correlations among *S100A9*, *SPRR2A*, and *FABP5* were also investigated. Finally, relationships between survival times and these genes were analyzed by GEPIA tools (http://gepia2.cancer-pku.cn/#index).

### qRT-PCR analysis

Primary cSCCs and patient-matched normal adjacent skin tissues for quantitative real-time PCR (qRT-PCR) were obtained from consecutive cSCC patients. Total RNA was extracted from cSCC tissues and paired adjacent normal skin tissue using Trizol reagent (Invitrogen, USA). Then, extracted RNAs were reverse-transcribed into cDNA and were then subjected to Taqman qPCR analysis on a 7900HT Fast RT-PCR System (Life Technologies, ThermoFisher, Loughborough, UK). The mRNA levels of *S100A9*, *SPRR2A*, and *FABP5* were detected by the SYBR Green qPCR system (Life Technologies) using the following primers (Supplementary Table S1). *GAPDH* mRNA level was used as a control. The relative expressions were calculated with 2^–ΔΔCT^ method.

### Immunohistochemistry staining

The detailed procedure for IHC staining was presented in our previous published study [24]. Briefly, samples were fixed in 10% neutral buffered formalin, dehydrated with gradient ethanol, hyalinized in xylene, embedded in paraffin, and cut into sections with a thickness of 5 μm. Sections were incubated with primary antibodies: anti-S100A9 (Abcam, #ab92507, 1:250) and anti-FABP5 (CST, #39926, 1:1200). The temperature was maintained at 4 °C overnight, followed by the co-incubation of secondary antibody for 1 h after washing with phosphate‐buffered saline. Finally, sections were immunostained using DAB plus kit.

### Cell culture and transfection

The human epidermoid carcinoma A431 cell line from the National Infrastructure of Cell Line Resource and SCL-1 cell line (Daixuan Biotech, Shanghai, China) were confirmed by the GENEWIZ commercial company (Suzhou, China). Then cells were seeded in cell culture dishes and cultured for 3 days till confluency at 90%. Small interfering RNA (siRNA) were provided by RIBOBIO (Guangdong, China). The small interfering negative control RNA (siNC) was nonhomologous to any human genome sequences. The siRNA-targeted sequences are present in Supplementary Table S2. All transfections were performed with Lipofectamine^®^ 3000 Transfection Reagent (Invitrogen) according to the manufacturer’s direction.

### CCK-8 assay

According to the standard manufacturer’s protocol, cell viability in different groups was tested by CCK-8 assay (Dojindo, Japan). A density of 5 × 10^3^ cells/well was seeded in 96‐well plates and incubated at 37 °C for different times (24, 48, and 72 h). Before testing, 10 μl of CCK-8 reagent was added to each well and for a 4 h incubation. After that, the absorbance (optical density) of cells was captured and recorded at 450 nm using a Varioskan Flash system (Thermo, USA).

### Cell apoptosis assay

Flow cytometer assay was used for detecting cell apoptosis. Briefly, cells were resuspended in fixation buffer and stained with 5 μl Annexin V/FITC and 5 μl propidium iodide (PI) solutions for 30 min, respectively. Then cells were analyzed by flow cytometry (BD Biosciences, USA) with FITC and PI channel.

### Wound-healing assay

Different groups of transfected cells were seeded in a 24-well plate and grew until almost 90% confluence. A 200 μl pipette tip was used to scratch a line on the cell layer. The movement of cells was captured by an Olympus microscope at 0 and 24 h, respectively. The mean distance of each wound was calculated by the Image J software.

### Western blot analysis

To investigate whether NF-κB pathway involving the cSCC progression, western blot (WB) analysis was performed to observe its change after *S100A9* or *FABP5* knockdown. The total protein was extracted from A431 and SCL-1 cell lines with RIPA buffer, and the concentrations were determined by a BCA protein assay kit (EpiZyme Biotechnology, Shanghai, China). Briefly, the equal amount of protein for each sample was separated by 12% SDS-polyacrylamide gels and transferred to the polyvinylidene difluoride membranes, which was then blocked in 5% skim milk for 1 h at room temperature, followed by incubating with the primary antibodies at 4 °C for one night. Antibodies against p65 (CST, #8242), p-p65 (CST, #3033), and GAPDH (CST, #5174) were diluted by 1:1000. After three times washing with tris-buffered saline tween buffer, the membranes were incubated with the secondary antibodies at room temperature for 1 h. Finally, the protein bands were visualized by the chemiluminescence method.

### Statistical analysis

Data were presented as the mean ± standard deviation (SD). Data were statistically analyzed using Prim 9. Statistical significance was determined by paired or non-paired Student’s *t*-test. *P* < 0.05 was considered statistically significant.

## Results

### Single-cell transcriptomes of cSCC

**Fig. 1:**
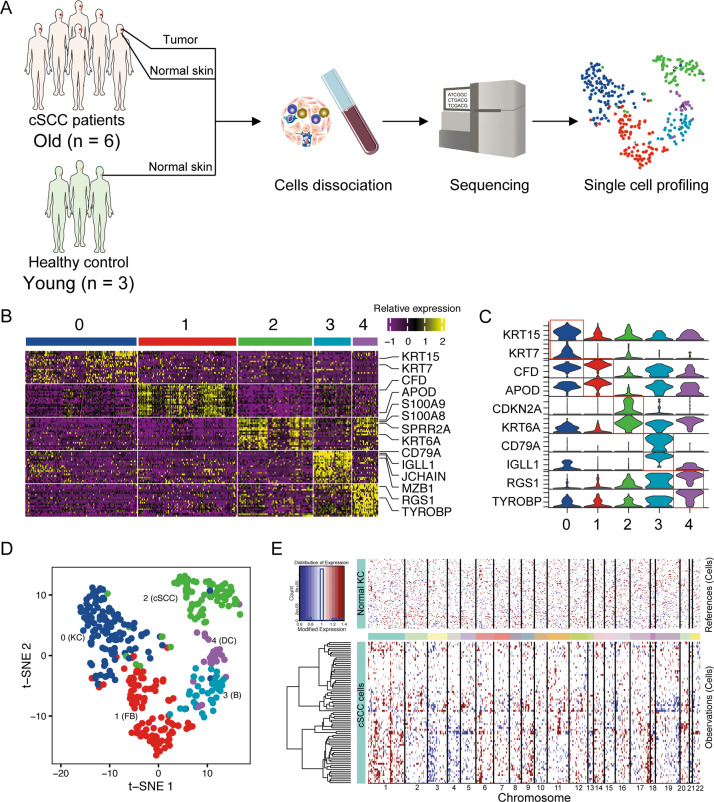
ScRNA-seq profiling of the cSCC tumor environments. **A** Workflow of cSCC patients and healthy control sample processing for scRNA-seq. **B** Heatmap showing the expression of representative genes in each cluster. The top bars label the clusters corresponding to specific cell clusters. **C** Violin plots displaying the expression of well-known representative markers across the cell types identified in cSCC. **D** T-distributed stochastic neighbor embedding (t-SNE) plot shows the annotation for cell types. **E** Heatmap showing large-scale CNVs of normal KC and cSCC cells. The normalized CNV levels were shown, the red color indicates high CNV level and blue indicates low CNV level.

Six cSCC tissues, paired adjacent skin, and three healthy control skin tissues were collected for scRNA-seq based on the Smart-seq2 strategy (Fig. 1A). After quality control, a total of 350 cells with an average of 6319 genes/cells were obtained for the following analyses. Then, five cell clusters consisting of normal KCs (112 cells), FBs (99 cells), cSCCs (75 cells), B cells (38 cells), and DCs (26 cells) after variable features selection, PCA and PCs selection (Fig. 1B–D). In general, most cells of the adjacent skin samples and healthy control skin samples were clustered into KC cluster and most cells of the cSCC tissues were clustered into cSCC cells and immune cells (Fig. 1D). Notably, three KCs from the cSCC adjacent tissues were clustered into the cSCCs cluster. Furthermore, no significant difference of KCs between the young and the old was found as they clustered into one cluster. Cluster-specific markers were identified to annotate cell populations. In most cases, widely used cell type markers were used for cell type definition. The cell markers are as follows: KCs: *KRT15*, *KRT7*; FBs: *CFD*, *APOD*; cSCC cells: *CDKN2A*, *KRT6A*; B cells: *CD79A*, *IGLL1*, *JCHAIN*, *MZB1*; DCs: *RGS1*, *TYROBP* (Fig. 1C).

### CNV landscape of cSCC cells

CNV analysis was often widely used to distinguish malignant from non-malignant cells. To investigate the malignancy of cSCC cells, we calculated large-scale chromosomal CNV in normal KC and cSCC cell clusters based on averaged expression patterns across intervals of the genome. We found that cSCC cells exhibited remarkably higher CNV levels than Normal KC (Fig. 1E). Specifically, most cSCC cells showed higher CNV levels on chromosomes 1, 3, 4, 6, 9, and 17. However, there were also some cSCC cells that showed low CNV scores.

### Dysregulated genes and pathways in cSCC

**Fig. 2:**
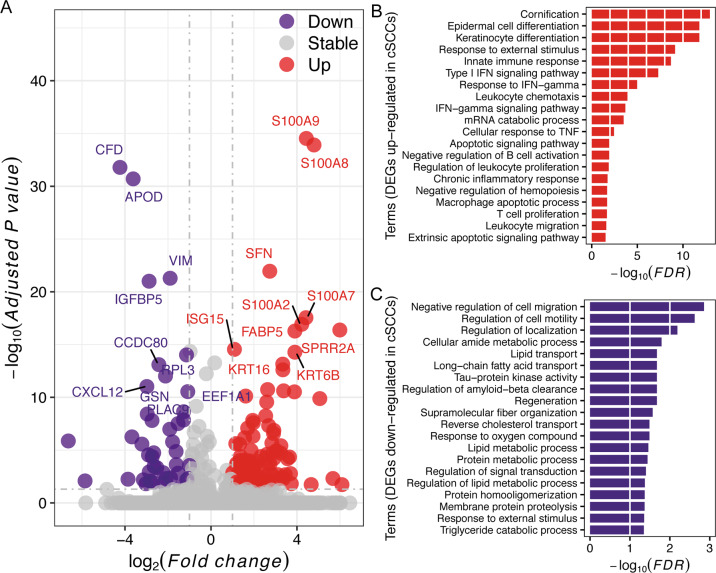
Dysregulated genes and biological processes in cSCC. **A** Volcano plot for DEGs identification between KCs and cSCCs. **B** Biological processes annotated by upregulated genes in cSCCs. **C** Biological processes annotated by downregulated genes in cSCCs.

In order to identify the DEGs between cSCC and normal KCs at a single-cell level, we performed differential expression analysis between KCs (Cluster 0) and cSCCs (Cluster 2) cells. A total of 161 DEGs were identified including 119 upregulated and 42 downregulated genes in cSCC (adjusted *P* value <0.05 and |logFC| > 1), which indicates that most of DEGs were upregulated in cSCC (Fig. 2A). The top 10 significantly upregulated genes were highlighted, including *S100A9*, *S100A8*, *SFN*, *S100A7*, *S100A2*, *SPRR2A*, *FABP5*, *ISG15*, *KRT6B*, and *KRT16*. In specific, seven keratin encoded genes (*KRT5*, *KRT6A*, *KRT6B*, *KRT6C*, *KRT14*, *KRT16*, and *KRT17*), six genes (*S100A2*, *S100A7*, *S100A7A*, *S100A8*, and *S100A9*) from the S100 family, and five genes (*SPRR2A*, *SPRR2B*, *SPRR2D*, *SPRR2F*, and *SPRR1B*) from SPRR family were significantly upregulated in cSCC cells, indicating the pivotal function of these gene families in cSCC progression. We also validated three upregulated genes including *S100A9*, *FABP5*, and *SPRR2A*, and three downregulated genes including *CFD*, *APOD*, and *VIM* by two external independent studies including one scRNA-seq study by 10X Genomics platform (GSE144240) and one bulk RNA-seq study (GSE108010). In general, the upregulation of *S100A9* and *FABP5*, and the downregulation of *CFD*, *APOD*, and *VIM* were validated in both public scRNA-seq study and bulk RNA-seq study, although the significant difference was not detected in all patients from the public scRNA-seq study (Supplementary Fig. S2). However, the upregulation of *SPRR2A* was only identified in one donor (patient 4) from the public scRNA-seq study (Supplementary Fig. S2A), and no difference was found in the bulk RNA-seq dataset (Supplementary Fig. S2B). The further qRT-PCR analysis also confirmed their expression (Supplementary Fig. S3).

GO annotation at the biological process level showed that most upregulated DEGs were annotated to immune responses such as, leukocyte chemotaxis, proliferation, and migration, together with the response to type I IFN, IFN-γ signaling pathway, and TNF signaling pathway (Fig. 2B). However, most downregulated DEGs were annotated to lipids metabolism (Fig. 2C). KEGG functional annotation showed that 12 significantly enriched pathways were identified (Supplementary Table S3), most of which were inflammation-related, i.e., IL-17 signaling pathway, and its downstream chemokine signaling and TNF signaling pathway.

### PPI network and GSEA analysis

**Fig. 3:**
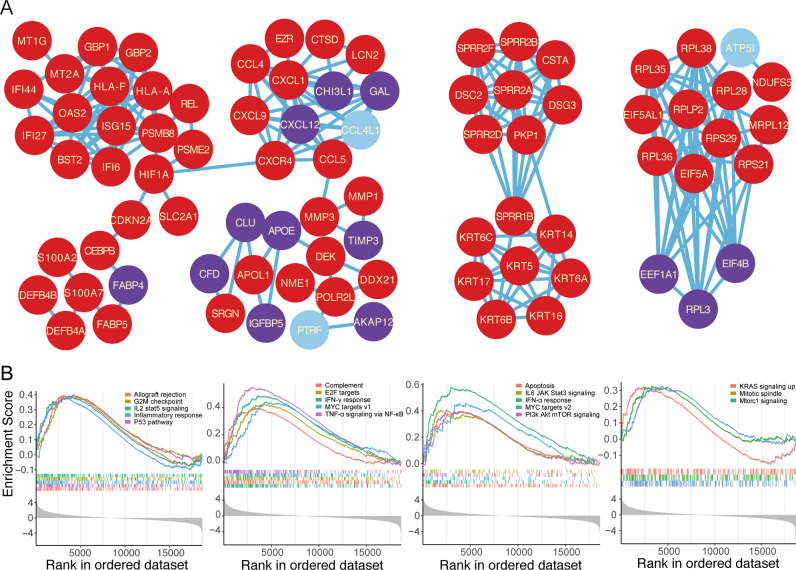
PPI network and GSEA analysis. **A** PPI network analysis shows the interactions among the DEGs. **B** Many cancer-related pathways were upregulated in cSCCs by GSEA.

Pathway annotation can reveal the related pathways in which the DEGs are involved, and PPI network analysis can demonstrate the potential interaction relationship. We found that most of the DEGs interact with many genes (Fig. 3A). Highly mutated oncogene *CDKN2A* interacted with *HIF1A*, which interacts with *CXCR4*. Meanwhile, *FABP5* connects with *FABP4*, which also interacted with *CDKN2A* by *CEBPB*. Another network is constructed by SPRR and KRT gene families. GSEA showed that 18 hallmarks of cancer were significantly enriched in cSCCs (Fig. 3B). Taken together, the S100 gene family, SPRR gene family, and *FABP5* may involve in cSCC progression.

### Positive correlation among *S100A9*, *SPRR2A*, and *FABP5*

**Fig. 4:**
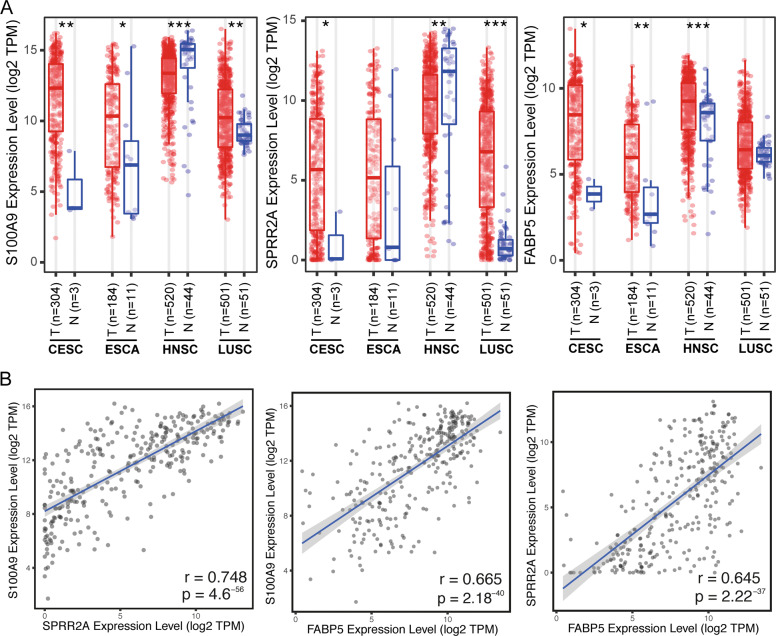
Expressions of *S100A9*, *SPRR2A*, and *FABP5* among different SCCs. **A** *S100A9*, *SPRR2A*, and *FABP5* expressions in CESC, ESCA, HNSC, and LUSC. **B** Spearman’s rank correlation between each other in CESC. cervical squamous cell carcinoma and endocervical adenocarcinoma for CESC, esophageal carcinoma for ESCA, head and neck squamous cell carcinoma for HNSC, and lung squamous cell carcinoma for LSCC. *P < 0.05, **P < 0.01, and ***P < 0.001.

As there is no cSCC-related RNA-seq data in the TCGA database, we investigated the expressions of *S100A9*, *SPRR2A*, and *FABP5* in four different kinds of SCCs including CESC, ESCA, HNSC, and LUSC. In general, these three genes were upregulated in CESC, ESCA, and LUSC (Fig. 4A). However, *S100A9* and *SPRR2A* were significantly downregulated in HNSC, and the expressions of *S100A9* and *SPRR2A* are very high in both HNSC tissues and normal tissue (Fig. 4A). Although the upregulation and downregulation of these genes in different SCCs should be further investigated, the positive correlations between each other are very high (Fig. 4B and Supplementary Fig. S4), indicating a tight relationship among these three genes. However, no significant effect on survival time was observed.

### *S100A9*, *SPRR2A*, and *FABP5* were overexpressed in cSCC tissues

**Fig. 5:**
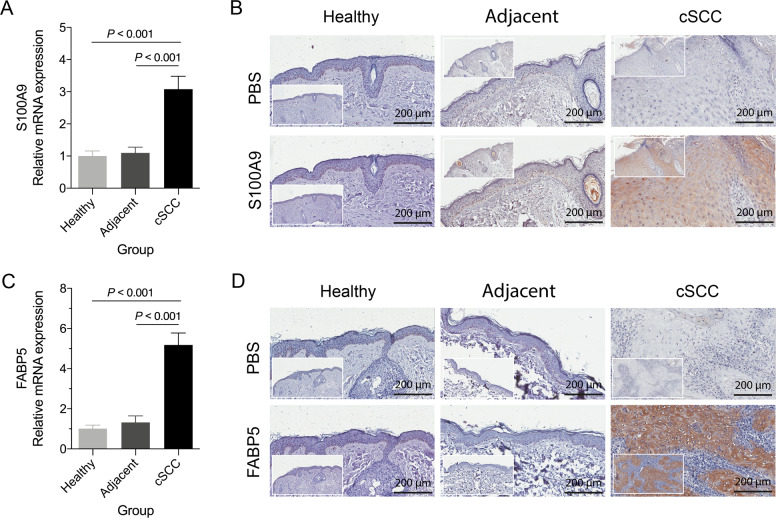
*S100A9* and *FABP5* were highly expressed in cSCC tissue. **A**, **B** *S100A9* was upregulated in cSCC tumor tissue at both mRNA level and protein level by qRT-PCR and IHC. **C**, **D** *FABP5* was upregulated in cSCC tumor tissue at both mRNA level and protein level by qRT-PCR and IHC. The statistical method is the paired Student’s *t*-test for the qRT-PCR results.

For the potential importance of S100, SPRR, and *FABP5* in cSCC progression, we further validated the expression of *S100A9*, *SPRR2A*, and *FABP5* by qRT-PCR and IHC in cSCC tissues, matched adjacent skin tissues, and normal skin tissues from healthy donors. qRT-PCR results showed that both of these three genes were significantly overexpressed in cSCC tissues at the mRNA level (*P* < 0.001, Fig. 5 and Supplementary Fig. S5), and there was no significant difference between healthy donors and adjacent skin tissues from cSCC (Fig. 5A, C). Since no significant effect on cell migration of *SPRR2A* in both A431 and SCL-1 cell lines, we did not validate its expression by IHC. Thus, the high expressions of *S100A9* and *FABP5* in cSCC tissue were confirmed by IHC (Fig. 5B, D). Collectively, these data further validate the expression of *S100A9* and *FABP5* in the mRNA and protein levels.

### *S100A9* and *FABP5* promoted cell proliferation and migration

**Fig. 6:**
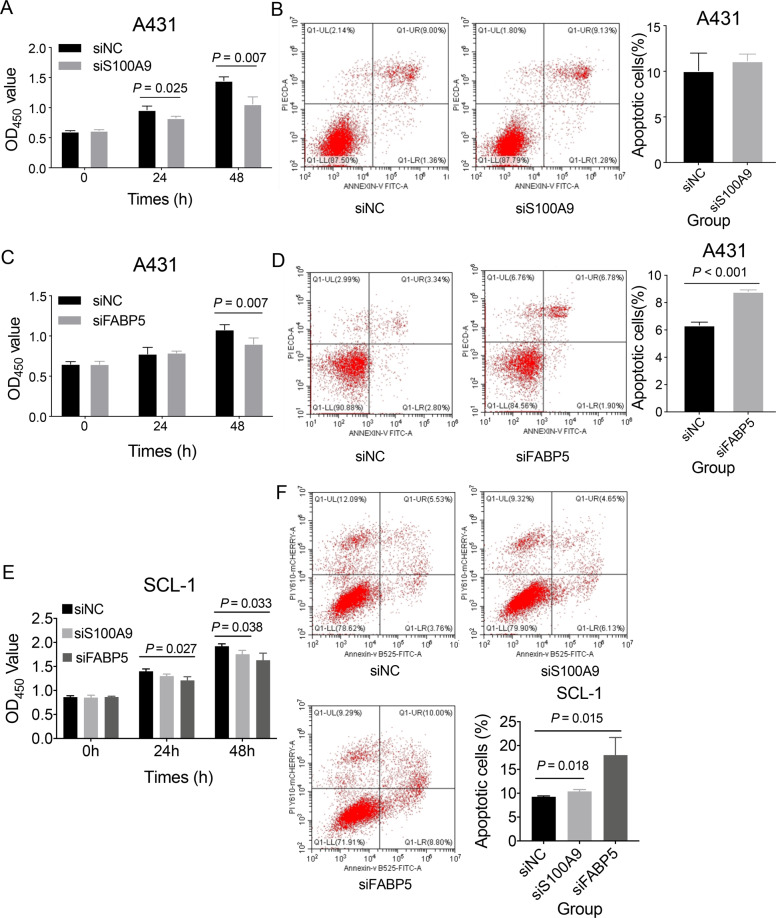
Effect of *S100A9* and *FABP5* on cell proliferation and apoptosis. **A** The effect of *S100A9* on A431 cell proliferation was determined by CCK-8. **B** The effect of *S100A9* on A431 cell apoptosis was measured by staining with Annexin V/PI, followed by FACS analysis. **C** The effect of *FABP5* on A431 cell proliferation was determined by CCK-8. **D** The effect of *FABP5* on A431 cell apoptosis was measured by staining with Annexin V/PI, followed by FACS analysis. **E** The effect of *S100A9* and *FABP5* on SCL-1 cell proliferation. **F** The effect of *S100A9* and *FABP5* on SCL-1 cell apoptosis. Data were presented as mean ± SD. siNC represents the siRNA negative control, and si*S100A9* and si*FABP5* represent the specific *S100A9* siRNA and *FABP5* siRNA, respectively.

**Fig. 7:**
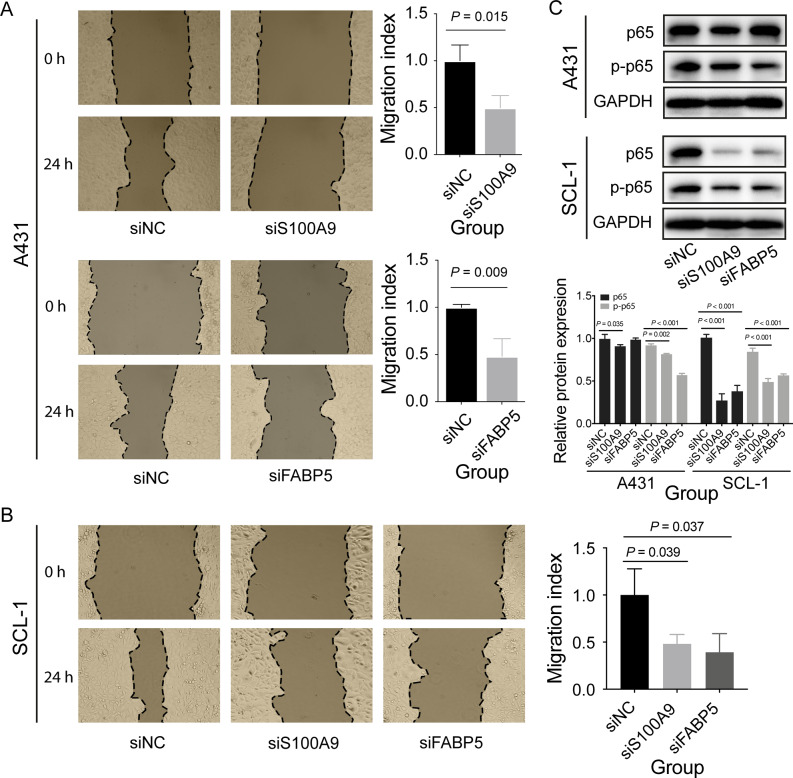
Effect of *S100A9* and *FABP5* on cell migration and NF-κB pathway. **A**, **B** The effect of *S100A9* and *FABP5* on cell migration and invasion were determined by wound-healing assay. **C** The effect of *S100A9* and *FABP5* on NF-κB pathway were determined by western blot analysis. Data were presented as mean ± SD. siNC represents the siRNA negative control, and si*S100A9* and si*FABP5* represent the specific *S100A9* siRNA and *FABP5* siRNA, respectively. The statistical method is the unpaired Student’s *t*-test.

To test whether *S100A9*, *SPRR2A*, and *FABP5* could contribute to the human cSCC cells progression, we investigated these genes on the migration and invasion effect of A431 and SCL-1 cell lines by knocking down their expressions using siRNA. These three genes were knocked down successfully confirmed by qRT-PCR (*P* < 0.05). CCK-8 assay showed that the ability of cell proliferation was significantly inhibited after 24 h when *S100A9* was interfered with siRNA (*P* < 0.05, Fig. 6). However, no significant apoptosis change was observed by the FACS instrument (Fig. 6). Regarding *FABP5*, we observed a significant decrease in cell proliferation (Fig. 6) and a significant increase of cell apoptosis (Fig. 6). No significant effect of *SPRR2A* on cell proliferation was observed (Supplementary Fig. S6). Furthermore, both *S100A9* and *FABP5* siRNAs groups exhibited a significant decrease in migration (Fig. 7A, B), while no significant decrease was found in the *SPRR2A* group (Supplementary Fig. S7). WB analysis showed that p-p65 expression was significantly decreased after *S100A9* or *FABP5* knockdown (*P* < 0.01, Fig. 7C), indicating that NF-κB pathway may involve the cSCC progression supervised by *S100A9* and *FABP5*.

## Discussion

cSCC serves as the second most common NMSC, caused by genetic mutations that result from endogenous and environmental factors, such as cumulative UV radiation. Numerous biological or genetic differences of tumor cells result in the cancer ITH [25], which plays a vital role in tumor progression and drug resistance [26]. Thus, it is important to thoroughly demonstrate the gene expression pattern of individual cells [27]. Single-cell transcriptome technology overcomes the disadvantages of traditional bulk RNA-seq methods, by profiling the whole-transcriptome profiles at a single-cell resolution and annotating different cell types from the tumor tissue [28]. Moreover, scRNA-seq will facilitate a clearer understanding of the molecular mechanisms in promoting tumor occurrence, and reveal the somatic mutations during tumor evolution [29]. In the present study, six primary cSCCs, patient-matched adjacent skin samples, and three healthy control skin tissues were used for scRNA-seq to decipherer the ITH of cSCC and driver genes in cSCC progression. A total of 350 high-quality cells were obtained, and five cell clusters were annotated after quality control. Regarding the normal KCs, there was no significant difference between the young and the old. However, three KCs from the cSCC adjacent tissues were also clustered into the cSCC cluster. In the present study, the cSCC patients were old, who undergo long-term UV radiation and whose adjacent skin was also aging. Thus, some KCs from the aged and UV-radiated adjacent or non-adjacent skin tends to harbor genetic mutation or epigenetic modifications, such as *TP53*, although the skin seems to be apparently normal [30]. Further CNV-based analysis confirmed the malignant of cSCC cells, as higher copy numbers were observed in cSCC cells.

Compared with the normal KCs, 119 upregulated and 42 downregulated genes were sought out in cSCC cells. Notably, many DEGs consisted of S100, SPRR gene family, including *S100A2*, *S100A7*, *S100A7A*, *S100A8*, *S100A9*, *SPRR2A*, *SPRR2B*, *SPRR2D*, *SPRR2F*, and *SPRR1B* were identified. In humans, the S100 protein family is composed of 21 members that exhibit a high degree of structural similarity but are not functionally interchangeable [31]. *S100A8* and *S100A9* are thought to be characteristic of several acute and chronic inflammatory disease, including psoriasis, inflammatory bowel disease, and rheumatoid arthritis [32]. Furthermore, *S100A8* and *S100A9* are upregulated in a variety of cancers such as lung cancer, prostate cancer, colon cancer, stomach cancer, and breast cancer [33]. However, decreased expression of *S100A8* and *S100A9* in Chinese esophageal squamous cell carcinoma were also reported [34]. Recent studies have shown that *S100A8*/*A9* was highly expressed in cSCC than that in normal and actinic keratosis tissues, and in vitro experiments confirmed that the overexpression of *S100A8*/*A9* could enhance the proliferation and invasiveness of cSCC [35, 36]. In the present study, we also detected overexpression of *S100A9* in cSCC tissues and validated it by two public datasets, qRT-PCR and IHC. *S100A9* knockdown inhibited the proliferation and migration of cSCC cell lines. However, there was no significant effect on cell apoptosis when downregulated *S100A9*. Regarding the involved pathways, studies have shown that *S100A8*/*A9* induces the activation of the NF-κB pathway, which increases the expression of *CXCL1*, *CCL5*, *CCL7*, and so on, whose products are known in angiogenesis, tumor migration, wound healing, and the formation of pre-metastatic niches into the surrounding tissue and organs [37]. We also observed the decrease of p-p65 after *S100A9* knockdown, which confirmed the NF-κB pathway in cSCC progression. Taken together, the S100 gene family, at least *S100A9*, played an important role in promoting cSCC progression, including proliferation and migration.

*SPRR2A* is one of SPRR gene that encodes for a skin cross-linking protein, which plays an important role in maintaining the structural integrity of the epidermal cornified cell envelope [38]. In the normal status, *SPRR2A* shows the highest expression in the cervix and esophagus, but with low or absent expression in mesenchymal tissues such as brain, heart, muscle, and adipose [39]. Besides, *SPRR2A* is dominantly overexpressed under inflammation, stress, and infection statuses to protect barrier epithelial cells of the skin, lung, and intestine [40, 41]. Until now, almost all studies focused on the cell process of *SPRR2A*-induced epithelial-mesenchymal transition (EMT), including embryogenesis, wound healing, and carcinogenesis [42, 43]. Although *SPRR2A* is commonly expressed in organs containing squamous epithelia, its expression mode is distinct among different SCCs. Specifically, *SPRR2A* is upregulated in LUSC [44], and HNSC [45], but downregulated in oral SCC [46], and ESCA [47]. Furthermore, the expression of *SPRR2A* is upregulated first, and then decreases with carcinoma progression (higher stage and lymphatic metastasis) in LUSC and HNSC [44, 45]. In the present study, we observed the overexpression of *SPRR2A* in primary cSCC cells and almost absent expression in normal KC, which is consistent with the results in LUSC and HNSCC. With regard to other cancers, *SPRR2A* is upregulated in gastric cancer [48], but downregulated in cholangiocarcinoma [49]. In the cholangiocarcinoma and liver cancer model, *SPRR2A* is activated by STAT-3, promoting EMT through the interaction with ZEB1 [39, 49]. In addition, *SPRR2A* can increase P53 deacetylation by impairing P300-P53 interactions to inhibit P53 transcriptional targets, accelerating EMT and wound healing [50]. Although down- and upregulation of *SPRR2A* were observed in different cancers, higher expression of *SPRR2A* will increase the local aggressiveness of cancers. In contrast, when cancers progress into poor differentiation or lymphatic metastasis, the expression of *SPRR2A* will decrease to give rise to metastases [44, 45, 49].

Dysregulated lipid metabolism is the most prominent metabolic alteration in various cancers, playing a vital role in their proliferation, invasion, metastasis, and response to different therapies [51]. *FABP5*, also known as epidermal FABP (E-FABP), is essential for KC homeostasis and differentiation, and maintenance of the skin-water permeability barrier by participating in fatty acid metabolism [52, 53]. In the current study, we identified *FABP5* was significantly upregulated in cSCC cells compared with the normal KCs, which is consistent with two studies that also confirmed the overexpression of *FABP5* by IHC in cSCC [54, 55]. After then, the same result was observed in actinic keratosis and Bowen’s disease [56], which are considered as two premalignant lesions of cSCC. Although many studies have reported the dysregulation of *FABP5* in cSCC or its premalignant lesions, no further experiment was conducted to elucidate its mechanism. Here, we found *FABP5* can promote cell proliferation, migration, and inhibit apoptosis by in vitro experiment, which was also reported in oral SCC [57]. Besides, *FABP5* promotes lymphatic metastasis in cervical cancer, prostate cancer progression, breast cancer progression, and KC differentiation by reprogramming fatty acid metabolism through the NF-κB signaling pathway [52, 58, 59]. Here, we also confirmed the suppression of NF-κB pathway after *FABP5* knockdown, underlining the importance of NF-κB pathway in cSCC progression again. In hepatocellular carcinoma, the upregulation of *FABP5* enhances hypoxia-inducible factor-1 alpha (*HIF-1α*) activity, causing lipid metabolism reprogramming, and carcinoma progression [60]. In addition, *FABP5* can capture *S100A7* from cytosolic psoriatic protein extracts to form the FABP5-S100A7 complex, and vice versa by overlay assays [61]. Collectively, *FABP5* is upregulated in various cancers, which promotes cancers progression or metastasis by reprogramming fatty acid metabolism through the NF-κB signaling pathway or enhancing *HIF-1α* activity. The specific mechanism of *FABP5* and NF-κB pathway in cSCC should be further investigated.

## Conclusion

In conclusion, our findings provide valuable resources for deciphering comprehensive gene expression landscapes of heterogeneous cell types in cSCC. In specific, many members from S100 gene family, SPRR gene family, and *FABP5* were highly expressed in cSCC cells. *S00A9* and *FABP5* influence cell proliferation and migration through NF-κB pathway. Thus, these results suggested that targeting *S100A9* or *FABP5* might be an effective approach for suppressing cSCC progression.

## Supplementary information

Author contribution

Supplementary information

## Data Availability

The data that support the findings of this study are available from the corresponding author upon reasonable request as another research is being conducted.

## Code availability

The complete computational analysis workflow of this study is available from the corresponding author upon reasonable request.
