# Supplementary material for: Single-cell transcriptomic analysis reveals the critical molecular pattern of UV-induced cutaneous squamous cell carcinoma
Source: Cell Death Dis. 2021 Dec 21;13(1):23. doi: 10.1038/s41419-021-04477-y (PMC8692455; doi:10.1038/s41419-021-04477-y)
Supplement: Supplementary file 1 — Author contribution [file 41419_2021_4477_MOESM1_ESM.pdf]

DECLARATION OF CONTRIBUTIONS TO ARTICLE

ADMC

Manuscript Number: CCDIS-21-2740

Journal Name: *Cell Death & Disease* (the 'Journal')

Proposed Title of the Contribution:

Single-cell transcriptomic analysis reveals the critical molecular pattern of UV-induced cutaneous squamous cell carcinoma (the 'Contribution)

Author(s):

Guorong Yan, Liang Li, Sibo Zhu, Yuhao Wu, Yeqiang Liu, Lude Zhu, Zijun Zhao, Fei Wu, Ning Jia, Caihe Liao, Long Jiang, Qingyu Zeng, Peiru Wang, Lei Shi, Zhe (the 'Authors')

For all *CDDis* articles, each person named as an author in the published version must be able to show he or she has contributed substantially to the article.

Authorship credit should be based on 1) substantial contributions to conception and design, acquisition of data, or analysis and interpretation of data; 2) drafting the article or revising it critically for important intellectual content; and 3) final approval of the version to be published. Authors should meet conditions 1, 2 and 3.

Any person who cannot be shown to have made a substantial contribution to the article cannot be listed as an author in the final version. The name of any person who is deemed to have made a minor contribution can, however, appear in the Acknowledgments section of the article.

Please complete the table below to indicate the contributions of all named authors to the manuscript.

Author Full Name:	Specification of Contribution to the Manuscript:
Guorong Yan	G.Y. performed formal analysis, visualization, wrote original draft and revised the manuscript
Liang Li	L.L. performed formal analysis and wrote original draft.
Sibo Zhu	S.Z. performed part of data analysis, validation, and wrote original draft
Yuhao Wu	Y.W. collected part of data and revised the manuscript.
Yeqiang Liu; Qingyu Zeng; Pei	Y.L., Q.Z., and P.W. provided the experiment resources.
Lude Zhu; Zijun Zhao	L.Z. and Z.Z validated some experiment result.
Fei Wu; Caihe Liao; Long Jiang	F.W., C.L., and L.J. performed investigation.
Ning Jia	N.J. collected part of data and revised the manuscript.
Zhe Zheng	Z.Z. performed data curation and revised the manuscript.
Shan Fang	S.F. collected part of data and revised the manuscript.
Guolong Zhang	G.Z. conceptualized, supervised this project, and revised the manuscript.
Yichen Tang	Y.T. conceptualized, supervised this project, and revised the manuscript
Xiuli Wang	X.W. conceptualized, supervised, administered this project, and revised the manuscript

Please complete the table below to indicate the contributions of all named authors to the figures.

Figure 1:

L.L. designed the panel A; L.Z., C.L., Z.Z., and L.J. collected the samples used in this study; G.Y. generated the left panels (B-E) and assembled them.

Figure 2:

G.Y. generated this figure and assembled it.

Figure 3:

G.Y. generated this figure and assembled it.

Figure 4:

L.L. generated this figure and assembled it.

Figure 5:

N.J. plotted panel A, F.W., Y.L, and Y.W. generated this figure; G.Y. assembled it.

Figure 6:

S.Z. generated this plot and S.Z. assembled it.

Figure 7:

S.Z. generated panel A and B; F.S. generated panel C; G.Y. assembled it.

Signed for and on behalf of the Author(s):	Print Name:	Date:
Xiuli Wang	Xiuli Wang	2021-12-01
