# Supplementary material for: Single-cell transcriptomic analysis reveals the critical molecular pattern of UV-induced cutaneous squamous cell carcinoma
Source: Cell Death Dis. 2021 Dec 21;13(1):23. doi: 10.1038/s41419-021-04477-y (PMC8692455; doi:10.1038/s41419-021-04477-y)
Supplement: Supplementary file 2 — Supplementary information [file 41419_2021_4477_MOESM2_ESM.docx]

**Supplementary information**

Single-cell transcriptomic analysis reveals the critical molecular pattern of UV-induced cutaneous squamous cell carcinoma

**Guorong Yan^1,^†, Liang Li^2,^†, Sibo Zhu^3,^†, Yuhao Wu^1^, Yeqiang Liu^4^, Lude Zhu^1^, Zijun Zhao^1^, Fei Wu^4^, Ning Jia^1^, Caihe Liao^1^, Long Jiang^2^, Qingyu Zeng^1^, Peiru Wang^1^, Lei Shi^1^, Zhe Zheng^1^, Shan Fang^1^, Guolong Zhang^1,^*, Yichen Tang^2,*^, Xiuli Wang^1,^***

^1^ Institute of Photomedicine, Shanghai Skin Disease Hospital, School of Medicine, Tongji University, Shanghai, 200092, China

^2^ Department of Dermatologic surgery, Shanghai Skin Disease Hospital, School of Medicine, Tongji University, Shanghai, 200092, China

^3^ State Key Laboratory of Genetic Engineering, School of Life Sciences, Fudan University, Shanghai, 200438, China

^4^ Department of Pathology, Shanghai Skin Disease Hospital, School of Medicine, Tongji University, Shanghai, 200092, China

**Figure Legends**

**
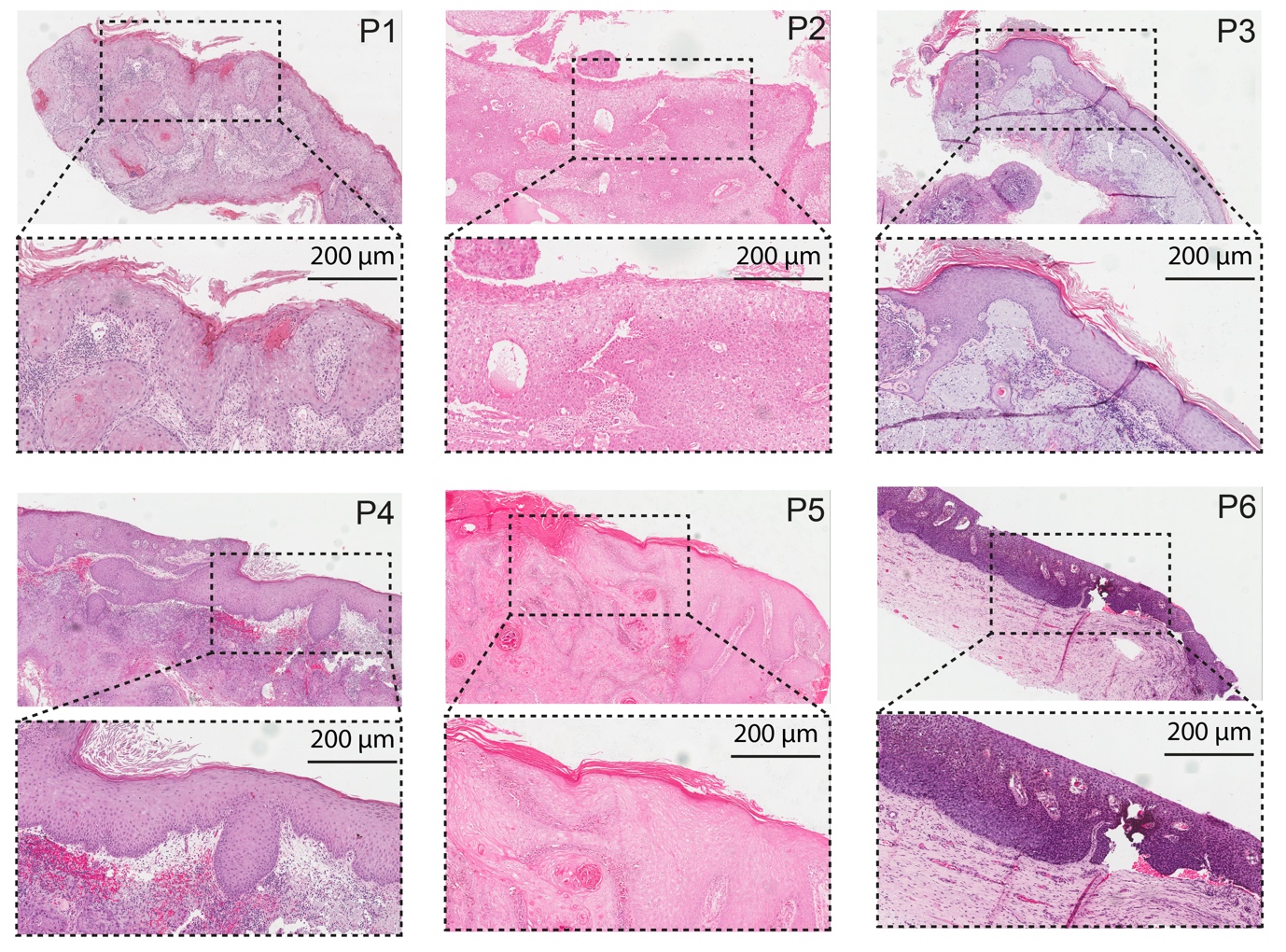
Fig. S1. Hematoxylin-eosin (HE) staining of the cSCC samples in the present study.**


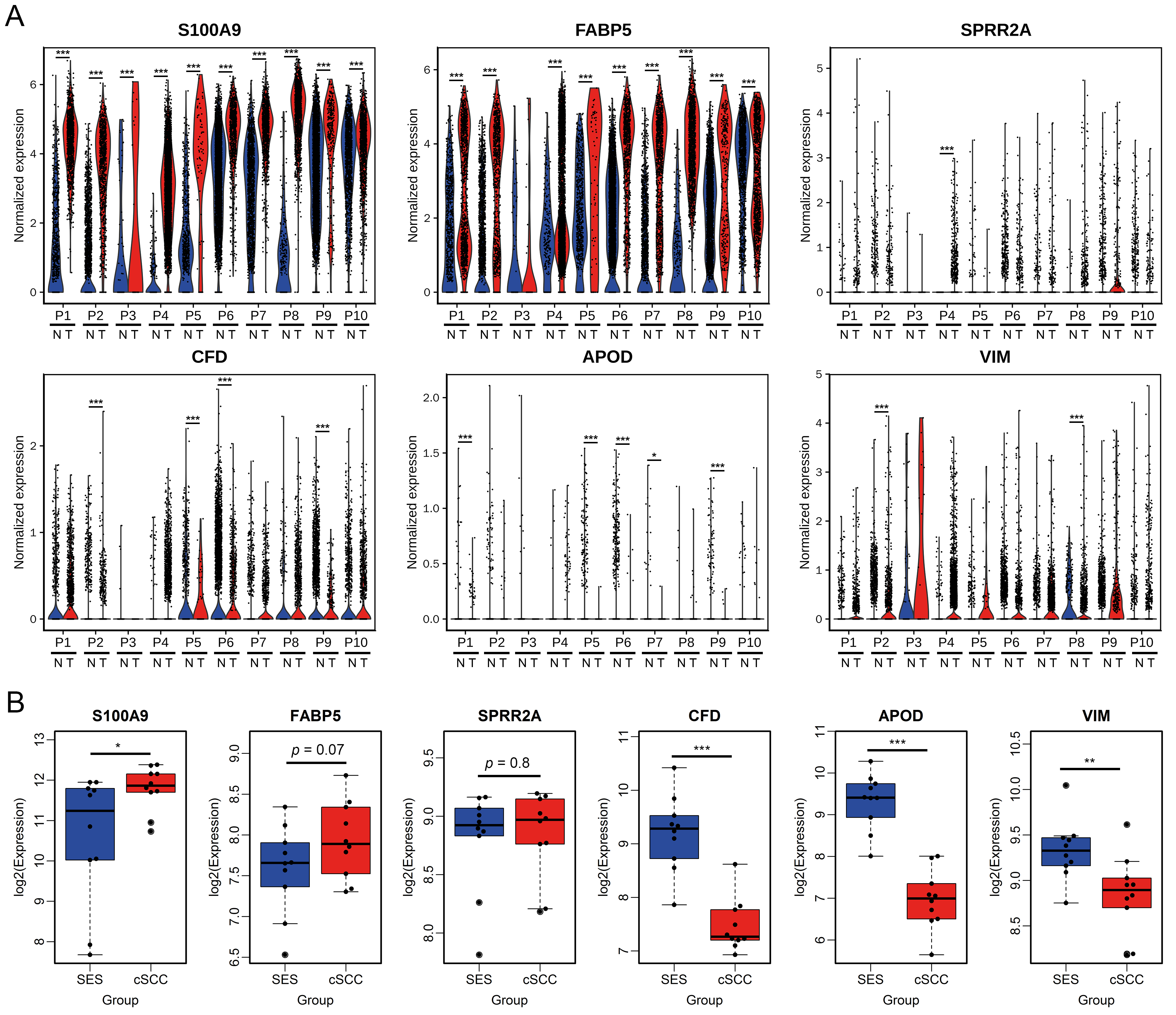


**Fig. S2. Gene expression validations by external scRNA-seq and bulk RNA-seq studies.** (**A**) from public scRNA-seq study with accession number GSE144240, (**B**) from public bulk RNA-seq study with accession number GSE108010.

**
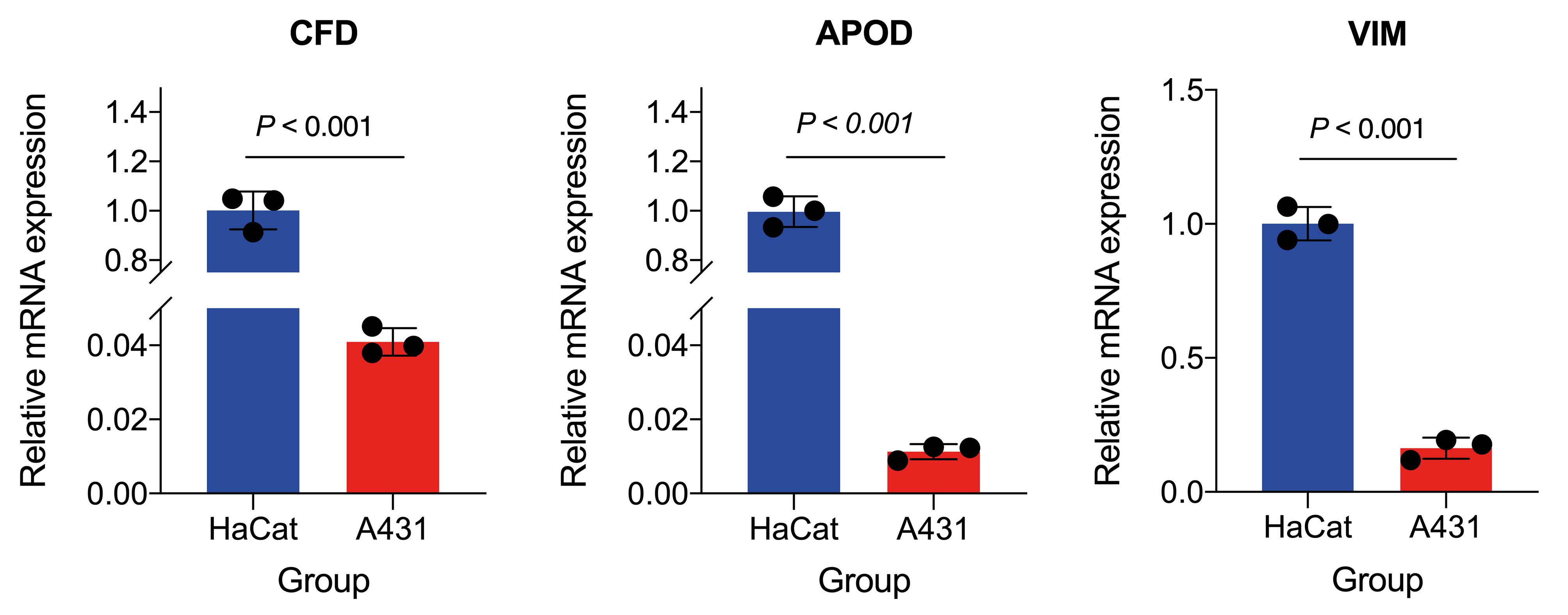
Fig. S3**. Down-regulated genes expression validated by qRT-PCR. Three down-regulated genes in cSCC cells were validated in A431 cell line.


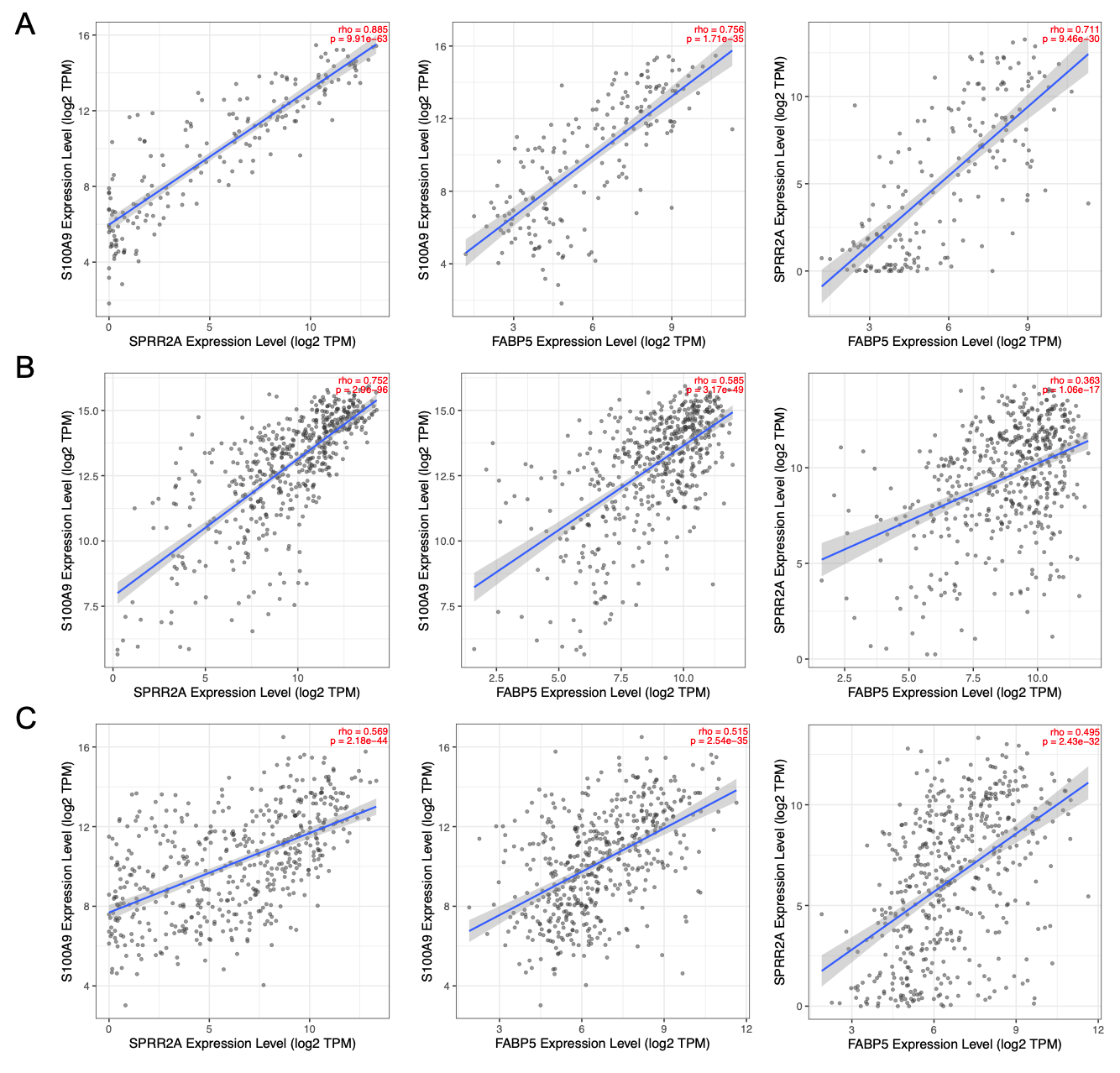
**Fig. S4. Spearman’s rank correlation between each other in ESCA (A), HNSC (B), and LUSC (C).** esophageal carcinoma for ESCA, head and neck squamous cell carcinoma for HNSC, and lung squamous cell carcinoma for LSCC.


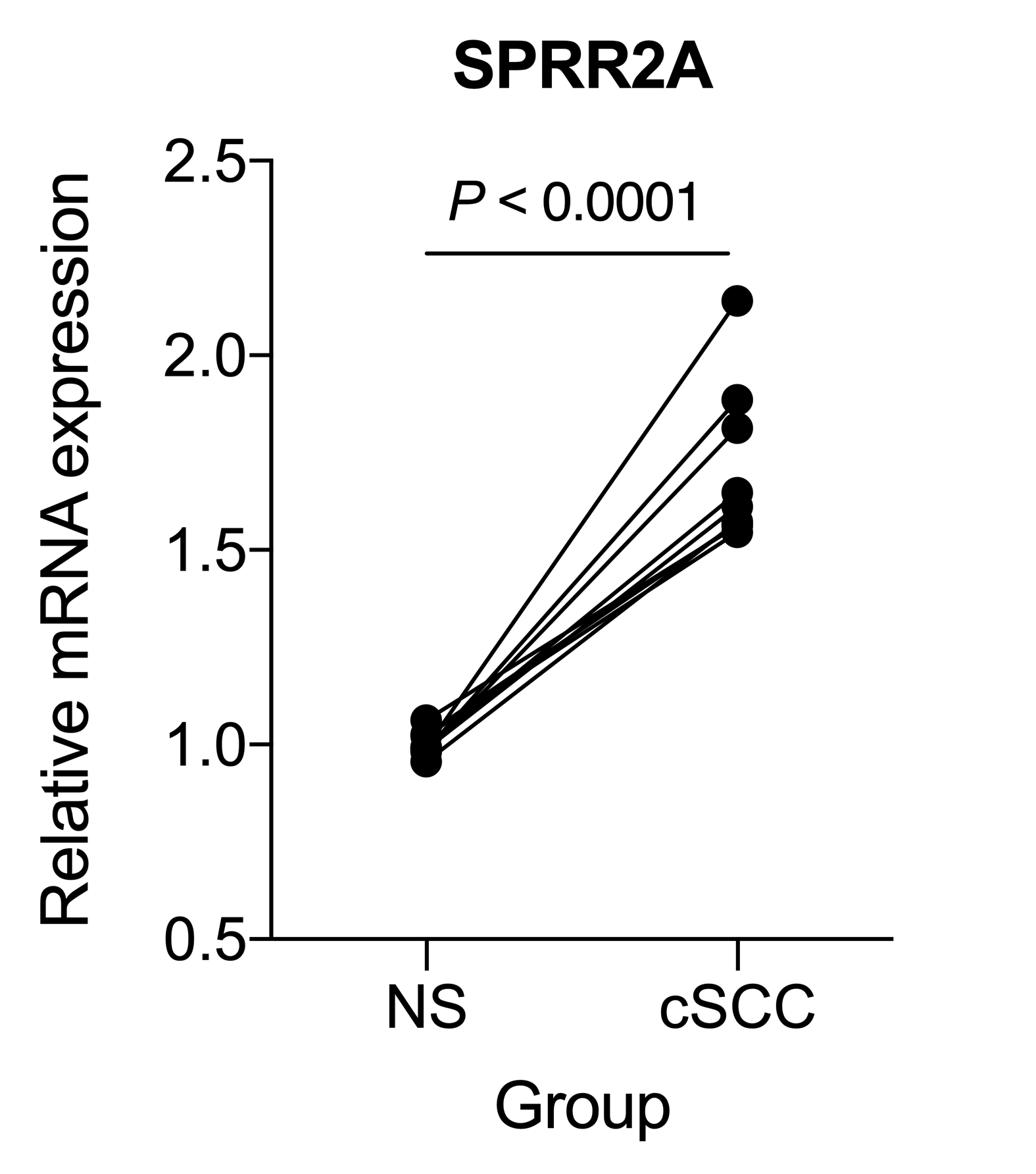
**Fig. S5. *SPRR2A* expression.** NS is adjacent tissues of cSCC samples.


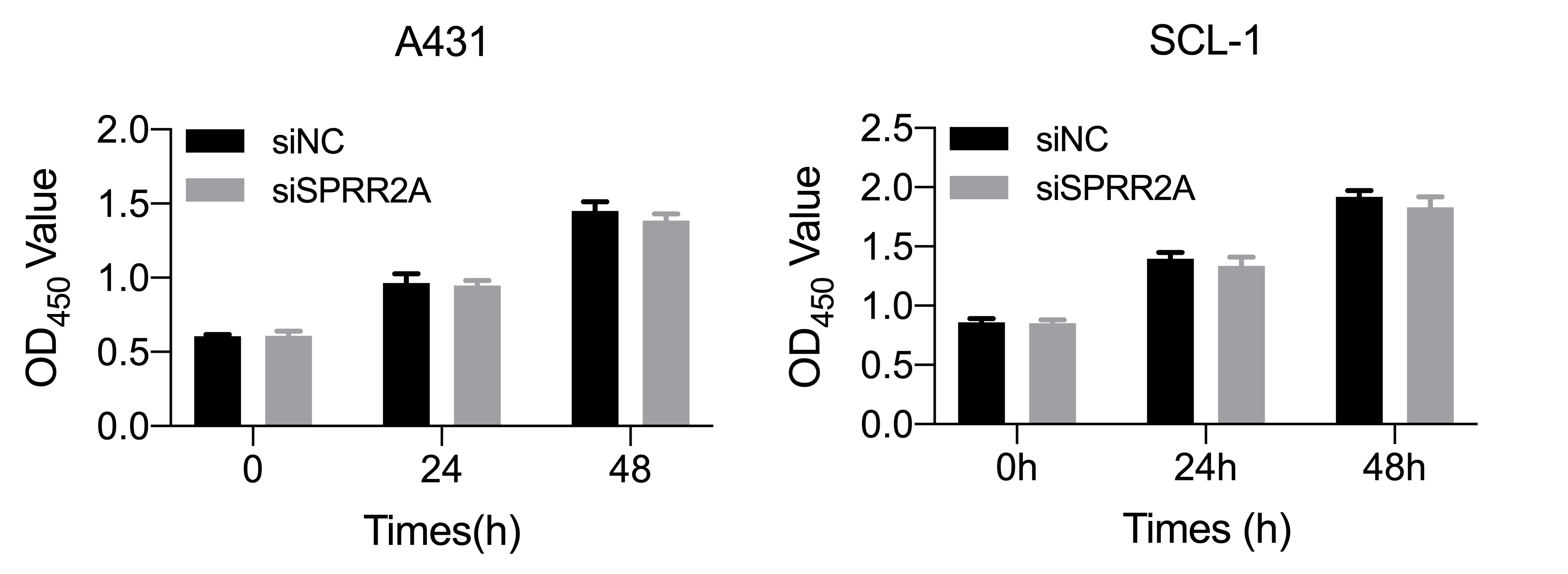


**Fig. S6. The effect of *SPRR2A* on cell proliferation was determined by CCK-8.**


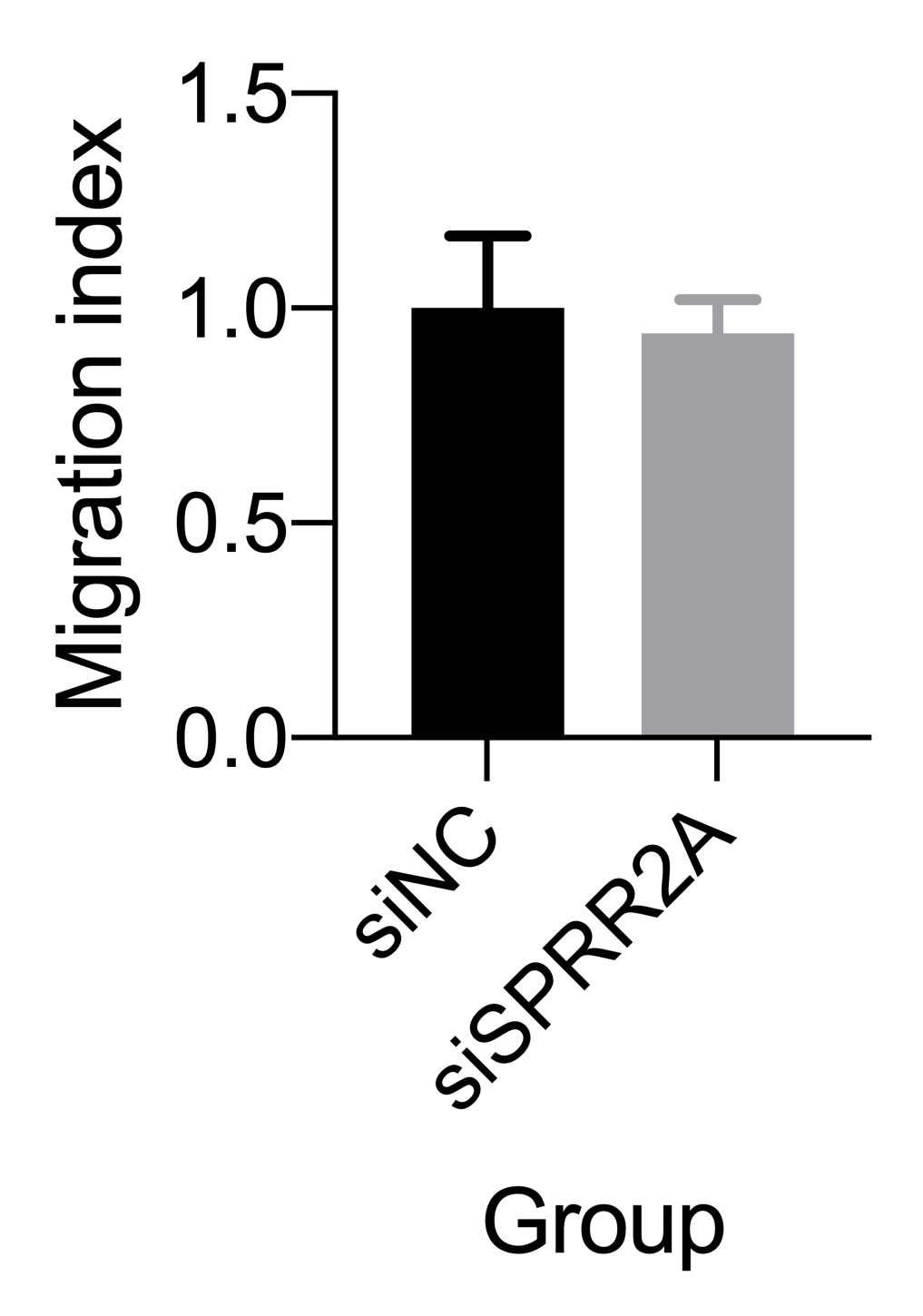


**Fig. S7. The effect of *SPRR2A* on A431 cell migration and invasion was determined by wound healing assay.**

**Tables**

**Table S1. Primer sequences for qRT-PCR.**

| **Gene** | **Forward sequence** | **Reverse sequence** |
| --- | --- | --- |
| *S100A9* | CATGCTGATGGCGAGGCTAA | CCACTGTGGTCTTAGGGGGT |
| *SPRR2A* | CAAAGGAAGTCCTGGGCTGT | TGATGGTTCCCAGGGAGAGA |
| *FABP5* | TGGCCAAGCCAGATTGTATCA | CTGATGCTGAACCAATGCACC |
| *CFD* | CACCATGCACAGCTGGGAG | AGGCCCAGGCCTCCTC |
| *APOD* | TTTATGCCATCGGCACCGTA | CCTGGTCTGTGACCGTCATT |
| *VIM* | TCCGCACATTCGAGCAAAGA | ATTCAAGTCTCAGCGGGCTC |

**Table S2. siRNA sequences**

| **Name** | **Sense sequence (5’-3’)** | **Antisense sequence (5’-3’)** |
| --- | --- | --- |
| *S100A9*-267 | GCUUCGAGGAGUUCAUCAUTT | AUGAUGAACUCCUCGAAGCTT |
| *SPRR2A*-243 | CACCCUGCCAGUCAAAGUATT | UACUUUGACUGGCAGGGUGTT |
| *FABP5*-206 | GGAGAGAAGUUUGAAGAAATT | UUUCUUCAAACUUCUCUCCTT |

**Table S3. Enriched pathways by KEGG analysis.**

| **ID** | **Description** | ***P* value** | **Involved genes** |
| --- | --- | --- | --- |
| hsa04657 | IL-17 signaling pathway | 1.3×10^-6^ | *MMP3*，*CEBPB*，*MMP1*，*S100A7*，*S100A8*，*S100A9*，*LCN2*，*CXCL1*，*DEFB4B*，*TNFAIP3* |
| hsa03010 | Ribosome | 0.0013 | *RPL35*，*RPL38*，*RPLP2*，*MRPL12*，*RPS21*，*RPL3*，*RPS29*，*RPL28*，*RPL36* |
| hsa04621 | NOD-like receptor | 0.0235 | *OAS2*，*GBP2*，*GBP1*，*TXN*，*CXCL1*，*CCL5*，*TNFAIP3* |
| hsa04060 | CCR interaction | 0.0265 | *INHBA*，*CXCL9*，*CXCL1*，*CXCL12*，*CXCR4*，*CCL5*，*CCL4*，*CCL4L1* |
| hsa04062 | Chemokine signaling | 0.0265 | *CXCL9*，*CXCL1*，*CXCL12*，*CXCR4*，*CCL5*，*CCL4*，*CCL4L1* |
| hsa04064 | NF-kappa B signaling | 0.0265 | *PLAU*，*CXCL12*，*TNFAIP3*，*CCL4*，*CCL4L1* |
| hsa04623 | Cytosolic DNA-sensing | 0.0265 | *POLR2L*，*CCL5*，*CCL4*，*CCL4L1* |
| hsa04668 | TNF signaling pathway | 0.0265 | *MMP3*，*CEBPB*，*CXCL1*，*CCL5*，*TNFAIP3* |
| hsa05169 | Epstein-Barr virus infection | 0.0265 | *HLA-F*，*POLR2L*，*PTMA*，*YWHAZ*，*HLA-A*，*VIM*，*TNFAIP3* |
| hsa05203 | Viral carcinogenesis | 0.0265 | *HLA-F*，*REL*，*MAPKAPK2*，*GSN*，*YWHAZ*，*HLA-A*，*CDKN2A* |
| hsa05206 | MicroRNAs in cancer | 0.0265 | *TIMP3*，*EZR*，*MCL1*，*PLAU*，*CDKN2A*，*VIM* |
| hsa05323 | Rheumatoid arthritis | 0.0265 | *MMP3*，*MMP1*，*CXCL1*，*CXCL12*，*CCL5* |
